# Integrative Assessment of Mixture Toxicity of Three Ionic Liquids on Acetylcholinesterase Using a Progressive Approach from 1D Point, 2D Curve, to 3D Surface

**DOI:** 10.3390/ijms20215330

**Published:** 2019-10-26

**Authors:** Huilin Ge, Shanshan Tao, Min Zhou, Bingjun Han, Hongqiu Yuan

**Affiliations:** 1 Hainan Key Laboratory of Tropical Fruit and Vegetable Products Quality and Safety, Analysis and Testing Center, Chinese Academy of Tropical Agricultural Sciences, Haikou 571101, China; sarah133@126.com (S.T.); zhoumin05@yeah.net (M.Z.); hanbjun@163.com (B.H.); yuanhongqiu@163.com (H.Y.); 2 College of Plant Science & Technology, Huazhong Agricultural University, Wuhan 430070, China; 3 College of Plant Protection, Hainan University, Haikou 570228, China

**Keywords:** ionic liquids, acetylcholinesterase, equivalent-surface, mixture toxicity, concentration addition, independent action, co-toxicity coefficient

## Abstract

The joint toxicities of [BMIM]BF_4_, [BMIM]PF_6_, and [HMIM]BF_4_ on acetylcholinesterase (AChE) were systematically investigated by using a progressive approach from 1D single effect point, 2D concentration-response curve (CRC), to 3D equivalent-surface (ES) level. The equipartition equivalent-surface design (EESD) method was used to design 10 ternary mixtures, and the direct equipartition ray (EquRay) design was used to design 15 binary mixtures. The toxicities of ionic liquids (ILs) and their mixtures were determined using the microplate toxicity analysis (MTA) method. The concentration addition (CA), independent action (IA), and co-toxicity coefficient (CTC) were used as the additive reference model to analyze the toxic interaction of these mixtures. The results showed that the Weibull function fitted well the CRCs of the three ILs and their mixtures with the coefficient of determination (*R*^2^) greater than 0.99 and root-mean-square error (RMSE) less than 0.04. According to the CTC integrated with confidence interval (CI) method (CTCICI) developed in this study, the 25 mixtures were almost all additive action at 20% and 80% effect point levels. At 50% effect, at least half of the 25 mixtures were slightly synergistic action, and the remaining mixtures were additive action. Furthermore, the ESs and CRCs predicted by CA and IA were all within the CIs of mixture observed ESs and CRCs, respectively. Therefore, the toxic interactions of these 25 mixtures were actually additive action. The joint toxicity of the three ILs can be effectively evaluated by the ES method. We also studied the relationship between the mixture toxicities and component concentration proportions. This study can provide reference data for IL risk assessment of combined pollution.

## 1. Introduction

Numerous facts have shown that humans and all other living organisms are exposed to chemical mixtures of multiple components in the environment [1]. The toxicity of pollutant mixture to organisms is manifested as joint toxicity [2]. Essentially, the evaluation of mixture toxicity interaction was based on CA and IA as an additive reference model [3]. Most of the mixture toxicity index can be derived from the CA model, such as the sum of toxic units (STU), additivity index (AI), similarity parameter (λ), mixture toxicity index (MTI) [4], and CTC [5]. CTC was a simple and effective method for evaluating the toxic interaction of mixtures such as pesticide mixture [5]. In this study, CTC would be used to evaluate the combined toxicity of mixtures on one-dimensional (1D) single effect point level. In a further step, the combined toxicity of mixtures was evaluated by comparing the observed CRCs and the predicted CRCs by CA and IA on two-dimensional (2D) curve level. In cartesian coordinate space, the CA were the isobole of straight form for binary mixture [6], and the ES of plane triangle form for ternary mixture [7]. Finally, the combined toxicities of mixtures were evaluated based on the ES containing the isobole on a three-dimensional (3D) surface level.

Ionic liquids (ILs) are organic salts with low melting points that have enormous potential for industrial use as green replacements for harmful volatile organic solvents (VOCs) [8]. Although ILs will not cause air pollution because of their negligible vapor pressures, some of them still present a non-negligible solubility in water, thus leading to aquatic environment risk [9]. Several studies reported the biological effects of ILs on the basis of different toxicological test systems such as enzymes, bacteria, algae, mammalian cells, plants, invertebrates, and vertebrates [10,11,12,13,14,15,16]. The mixtures of ionic liquids are increasingly applied in practical applications such as solvents for chemical synthesis and process chemistry, electrochemistry, chromatography, and heat transfer fluids [17]. It is of great importance to predict and assess their combined effect before the likely industrial release into the environment. A few studies reported the combined effects of ILs. For example, the mixture effects of imidazolium ILs on *Triticum aestivum* and *Scenedesmus vacuolatus* [18]. Imidazolium ILs can cause multiple toxicity interaction in different composition and concentration range [19]. The synergism and antagonism of imidazolium ILs are well related to the concentration ratio of ILs with BF_4_^−^ [20].

AChE is a key enzyme in nervous system and is most commonly used in enzyme inhibition method [21]. The AChE inhibition method had been used to characterize the toxicity of pollutants such as organophosphorus and carbamate pesticides, and ILs [10]. Stolte et al. studied the (eco)toxicity and biodegradability of ILs based on AChE test [22]. Yan et al. predicted the toxicity of ILs on AChE by the quantitative structure–activity relationship (QSAR) method using topological indexes [23]. Cho et al. interpreted the toxicological activity of ILs on AChE via in silico modeling [24]. Zhu et al. predicted the toxicity of ILs on AChE using QSAR models of multiple linear regression and extreme learning machine algorithms [25].

In the present study, three widely used imidazole ILs including [BMIM]BF_4_, [BMIM]PF_6_, and [HMIM]BF_4_ were selected as the mixture components. The binary mixtures were designed by the EquRay method, the ternary mixtures were designed by the EESD method, and the toxicities of single ILs and their mixtures were determined using the AChE-based MTA method [26]. The mixture toxicity interaction was evaluated on 1D point, 2D curve, and 3D surface levels.

## 2. Results and Discussion

### 2.1. Single Toxicity

**Figure 1:**
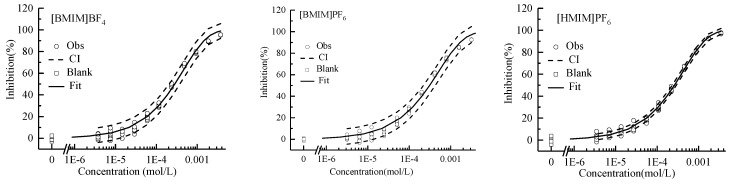
Concentration–response curves of single ILs inhibiting AChE. Square: blank control; Circle: observed data; Solid line: Weibull model fit; Dashed line: confidence interval.

The regression models and the estimated parameters of the toxicity of single ILs on AChE were summarized in Table 1 and the resulting CRCs were visualized in Figure 1. The CRCs were all S-type curves and can be well fitted by two-parameter Weibull function with RMSE < 0.04 and *R*^2^ > 0.99, indicating good correlation relationships between the exposure concentrations of ILs and the inhibition effects. The variability of the blank control in the test was controlled within ±10%. The indicators of effect concentration EC_80_, EC_50_, and EC_20_ were shown in Table 1. According to these indicators, the toxicity order of single ILs was [HMIM]BF_4_ > [BMIM]BF_4_ > [BMIM]PF_6_. Both the present study (AChE) and a previous study (luciferase) [27] all indicated that the ILs with BF_4_^−^ anions showed higher enzyme inhibitive toxicity. Moreover, ILs with BF_4_^−^ anions were also inclined to produce higher toxicity on other organisms such as the luminescent bacterium Q67 [28] and MCF-7 mammalian cells [13]. On the other hand, ILs with long alkyl chains showed higher AChE inhibitive toxicity. Similar results can be found in the report [29]. Egorova and Ananikov reviewed the main factors modulating the toxicity of ILs [30].

### 2.2. Mixture Toxicity Assessment on 1D Point Level

The EC_80_, EC_50_, and EC_20_ values for ternary and binary mixtures were listed in Table 1. According to Equation (5) using the *EC_x_*_,*i*_, *EC_x_*_,mix_, and *P_i_*, the CTC of IL mixtures can be obtained as shown in Table 2. According to the CTC judgment criteria in ref. [31], at 50% and 80% effect levels, the mixtures overall presented additive action, except for the synergistic action of G8, B3, B4, B6, B8, and B11 at 50% effect and B6 and B7 at 80% effect. At 20% effect, the mixtures overall presented synergistic action, except for the additive action of G0, G1, G3, G4, B5, B7, B9, B13, and B14. However, conclusions based on data without uncertainty were unreliable to a certain extent.

According to the CTCICI method developed in this study, mixtures of G0–G9 and B1–B15 were almost all additive action at 20% and 80% effect levels, except for the slightly synergistic action of G8, B3, and B12 at 20% effect, and B6, B7, and B12 at 80% effect. For mixtures of G0–G9 and B1–B15 at 50% effect level, G1, G2, G5–G8, B3, B6–B8, and B10–B12 were slightly synergistic action, the remaining mixtures were additive action. The interaction types judged by the two methods were different, especially for mixtures at 50% effect level. The main reason was the difference in judgment criteria that one was based only on the observed CTC, another was based only on the observed CI of CTC.

We compared the conclusions judged based on the CTCICI method with those judged based on the model deviation ratio (MDR) method [32] and the combination index integrated with CI method [33], which were consistent with each other. Theoretically, this result can be obtained.

### 2.3. Mixture Toxicity Assessment on 2D Curve Level

**Figure 2:**
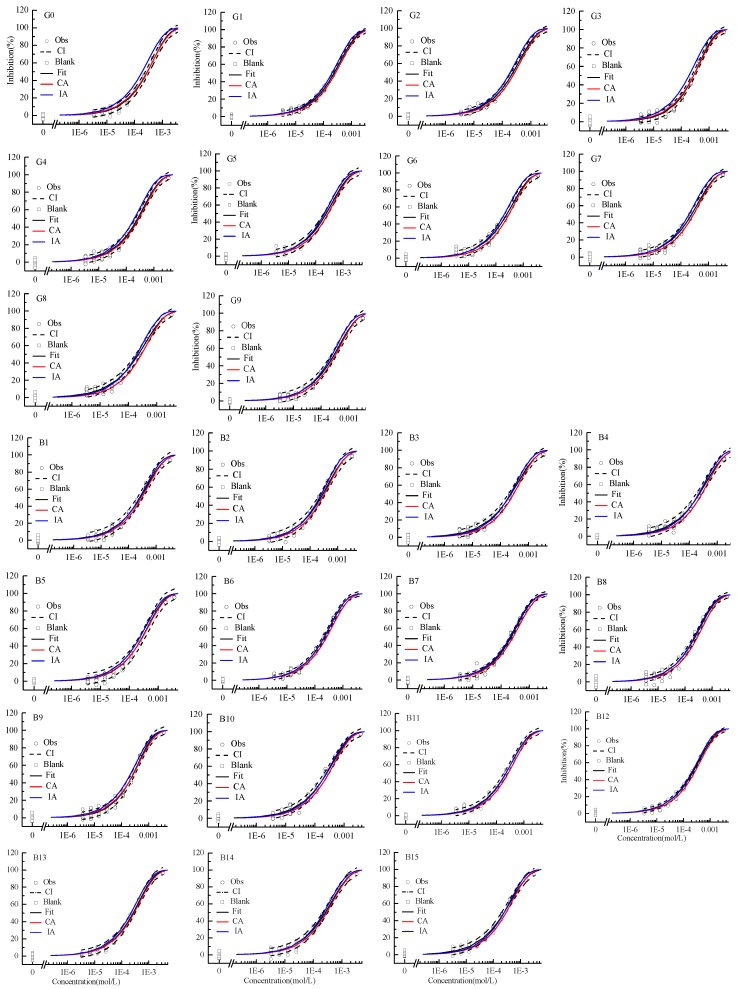
Concentration–response curves of ternary and binary mixtures of ILs inhibiting AChE. Note: Square: blank control; Circle: observed data; Black solid line: Weibull model fit; Red line: CA prediction; Blue line: IA prediction; Black dashed line: confidence interval.

The mixture CRCs predicted by CA and IA together with the experimental data and the fitted curves were integrated and displayed in Figure 2. These CRCs can also be depicted by the Weibull function. In all cases, the *R*^2^s were greater than 0.99 and the RMSEs less than 0.03, exhibiting good fitting ability.

The observed CRCs of G0, G3, B5, and B9 mixtures well agreed with CA prediction. The observed CRCs of B6–B8, B10, and B11 mixtures well agreed with IA prediction. Overall, the CRCs predicted by CA and IA models were all within the CI of mixture observed CRCs, the ternary and binary mixtures of [HMIM]BF_4_, [BMIM]BF_4_, and [BMIM]PF_6_ presented additive action on AChE.

**Figure 3:**
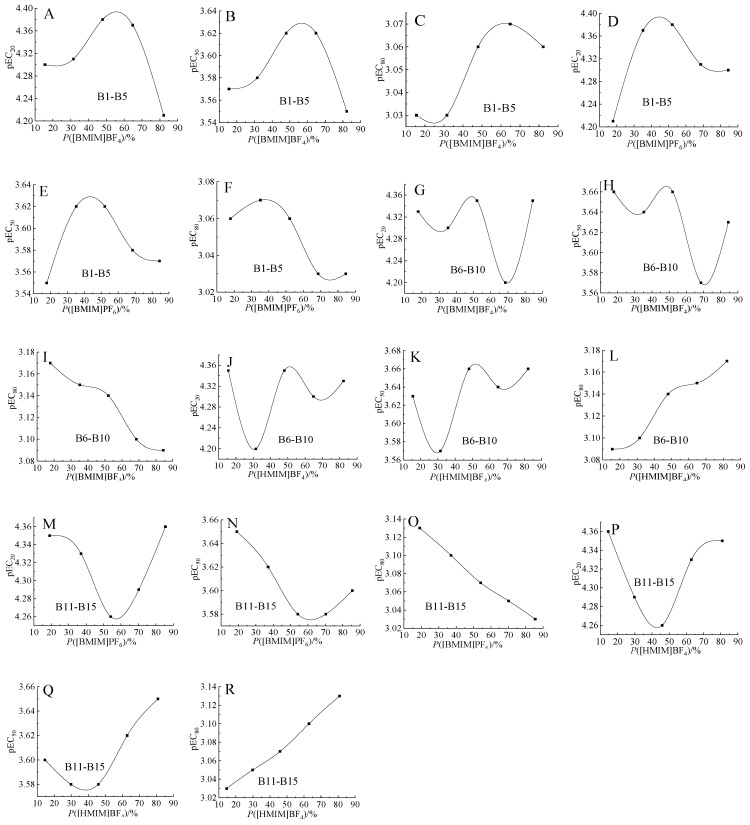
Relationship between the toxicity (pEC*_x_*) of binary mixtures and the concentration proportion of components. (**A**–**F**): mixtures of [BMIM]BF_4_ and [BMIM]PF_6_; (**G**–**L**): mixtures of [BMIM]BF_4_ and [HMIM]BF_4_; (**M**–**R**): mixtures of [BMIM]PF_6_ and [HMIM]BF_4_.

Studies indicated that there may be a correlation relationship between the mixture toxicity and the concentration proportion (*P_i_*) of components, for example the linear relationship in reference [19]. In the present study, there were two pairs of relatively obvious linear relationship between the mixture pEC_80_ and the *P_i_* of single ILs, i.e., B6–B10 ([BMIM]BF_4_ and [HMIM]BF_4_ mixtures) shown in Figure 3I,L and B11–B15 ([BMIM]PF_6_ and [HMIM]BF_4_ mixtures) shown in Figure 3O,R. For these mixtures in each pair relationship, the more toxic component presented monotonically increasing relationship, the less toxic component presented monotonically decreasing relationship. Similarly, in the mixtures including 12 components, a monotonically increasing relationship between mixtures pEC_50_ and the *P_i_* of the most toxic component was also observed [34].

Secondly, the inverted U-shaped relationship was observed between B1–B5 mixture toxicities (pEC_20_, pEC_50_, pEC_80_) and the *P_i_* of [BMIM]BF_4_ or [BMIM]PF_6_ shown in Figure 3A–F. Thereinto, the smallest mixture toxicities (pEC_20_, pEC_50_) were observed in mixture B5 as shown in Table 1 and Figure 3A,B, when [BMIM]BF_4_ having more toxic effect presented the largest *P_i_*. The U-shaped relationship was observed between B11–B15 mixture toxicities (pEC_20_, pEC_50_) and the *P_i_* of [BMIM]PF_6_ or [HMIM]BF_4_ as shown in Figure 3M,N,P,Q. This type of U-shaped or inverted U-shaped curve between mixture toxicities and component *P_i_* conformed with the climax hypothesis proposed by Lin et al. [35], i.e., the highest or lowest point of the curve was usually at the equitoxic ratio. The linear or biphasic relationships may reflect some differences in the mechanism of action.

Finally, no obvious correlation relationship was observed between B6–B10 mixture toxicities (pEC_20_, pEC_50_) and the *P_i_* of [BMIM]BF_4_ or [HMIM]BF_4_ as shown in Figure 3G,H,J,K. So did the ternary mixtures. Therefore, whether there must be a monotonic or biphasic relationship that occurs in more complex and more component mixtures needs to be further studied.

### 2.4. Mixture Toxicity Assessment on 3D Surface Level

**Figure 4:**
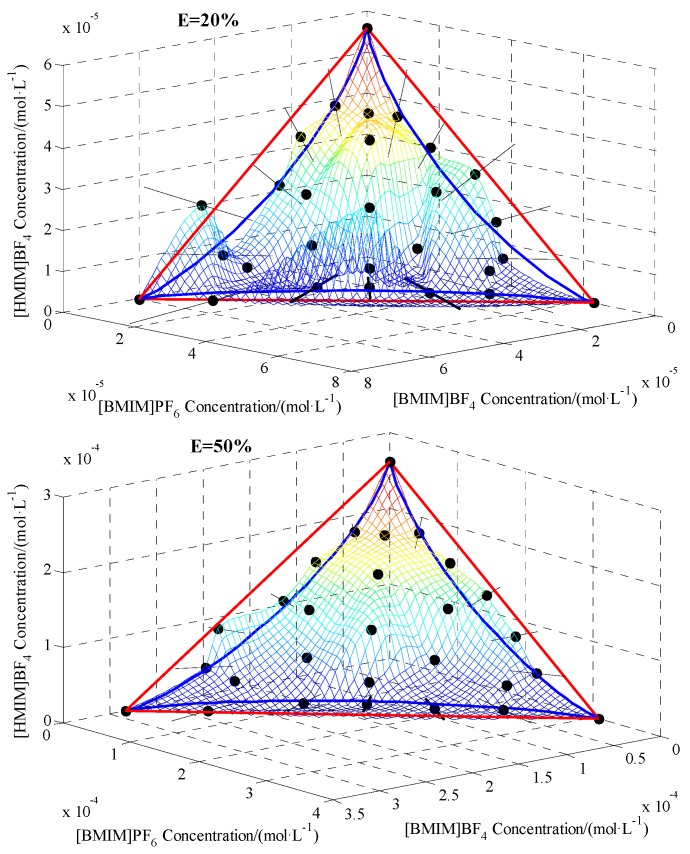

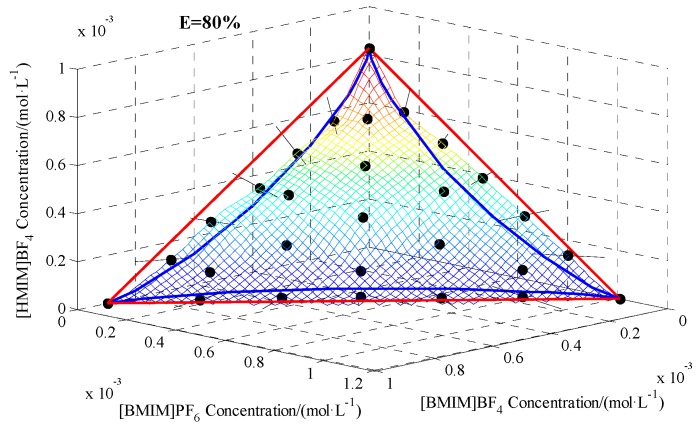
Equivalent-surface of ILs mixtures at 20%, 50%, and 80% effect levels. Note: Black point: observed point; Black line: confidence interval of observed point; Grid: observed equivalent-surface (ES); Red: CA ES; Blue: IA ES.

Figure 4 showed the front view of the ES of ILs mixtures at 20%, 50%, and 80% effects, which were composed of mixture observed ES (grid surface), CA ES (red triangular plane), and IA ES (blue triangular surface). The three edges of CA ES were the isobole of binary mixtures. The 28 black points were the observed equivalent points from binary mixtures (B1–B15), ternary mixture (G0–G9), and single ILs. The black lines on both sides of the black dot were their 95% CI.

As can be seen, due to the design of EESD, the mixture points are evenly distributed on the ES. The mixture observed ESs at 20%, 50%, and 80% effect levels were well located within the additive space formed by the IA ES and CA ES. Therefore, these mixtures were additive. Comparatively speaking, the variation of mixture observed ES at 20% effect appeared to be relatively large. At least half of the mixtures at 50% effect level was synergistic action judged by the CTCICI method in Section 2.2, which can be well explained here, because the CI of mixture observed ES was lower than the CA plane. However, the CI of mixture observed ES was still within CA ES and IA ES, so this type of synergistic was actually considered as additive. To avoid qualitative error, CA and IA should be used in combination to comprehensively judge the toxicity interaction of mixtures.

Why did these ILs cause additive action on AChE, which may be explained from the structural similarity. The cationic part of ILs played an important role in inhibiting the enzyme [36]. The structure of the positively charged imidazole ring was similar to that of choline which bound to AChE anion site [29]. The cations of ILs may bind to AChE anionic site with negative charge to inhibit the enzyme. So, the three ILs may have similar mechanism of action on AChE, and they showed additive action on AChE.

In the present study, when all the Weibull *b* values of the three ILs were less than 2.3, the IA ES was under the CA ES. Similar result can be found in the report [7]. It can be predicted that when all the Weibull *b* values of three components are 2.3, IA ES will coincide with CA ES. When the Weibull *b* values of three components are greater than 2.3, IA ES will be above CA ES. This hypothesis is worthy of further experimental verification.

The combined action of three ILs on AChE at different effect levels can be intuitively and effectively evaluated by the ES and EESD method. In addition to the function of optimal design of component mixing, EESD method also provides a way to construct the IA ES. It should be noted that the ES is different from the concentration–response surface (CRS) of binary mixtures [37]. The advantages of ES and EESD are that the toxicity interactions of three components can be shown in a comprehensive graph of ES, and EESD has definite spatial geometric significance. The disadvantages of ES and EESD are that for four or more component mixture, ES cannot be shown, and EESD cannot be applied. In this case, uniform design and other methods should be adopted [38].

## 3. Materials and Methods

### 3.1. Chemicals

The IL components included 1-butyl-3-methylimidazolium tetrafluoroborate ([BMIM]BF_4_), 1-butyl-3-methylimidazolium hexafluorophosphate ([BMIM]PF_6_), and 1-Hexyl-3-methylimidazolium tetrafluoroborate ([HMIM]BF_4_). The chemical structures and related information of acetylthiocholine iodide (ATCI), 5,5′-Dithio-bis(2-nitrobenzoic acid) (DTNB), AChE (*Electrophorus electricus*), and these ILs were shown in Table 3.

The stock solutions of ATCI, DTNB, AChE, [BMIM]BF_4_, [BMIM]PF_6_, and [HMIM]BF_4_ were separately prepared through dissolving them in the phosphate buffer (pH = 6.8, including 0.025 mol/L KH_2_PO_4_ and 0.025 mol/L Na_2_HPO_4_·12H_2_O) and stored in 4 °C refrigerator, and 0.379 g of sodium bicarbonate was added in 1 g DTNB [21]. The stock solutions of IL mixtures were prepared through mixing the stock solutions of individual ILs according to their concentration ratios assigned.

### 3.2. Acetylcholinesterase Toxicity Test

The toxicities of single ILs and their mixtures were expressed as percentage inhibition of the AChE colorimetric system. According to the methods of MTA [39] and colorimetric determination of AChE [21], we established a method of AChE-based MTA [26].

IL chemicals and their mixtures with 11 concentration series in triplicates and 8 controls were arranged in a microplate as follows: 100 µL phosphate buffer (pH = 6.8) was added to 8 wells in the twelfth column as blank controls, 100 µL of the solutions of IL chemicals and their mixtures with 11 gradient concentrations according to a geometric dilution factors of 0.5 were added to the wells from the first to the eleventh column. Then, 50 µL DTNB of 1 g/L, 50 µL ATCI of 1 g/L and 50 µL AChE of 0.2 U/mL were added into each test well to reach the final test volume of 250 µL. The absorbance of the AChE system exposed to single ILs and their mixtures were determined on Synergy 2 Multi-Mode Microplate Readers (BioTek Instruments, Winooski, VT, USA) with a 96-well white flat bottom microplate (Corning 9018). The absorbance in the microplate wells were determined in 412 nm after 0 min and 15 min of exposure at (29 ± 1) °C.

The effect (*E* of *x*%) of individual ILs and their mixtures was calculated as Equation (1). The CRCs were fitted by Weibull function shown in Equation (2) using least squares method [3]. The goodness of fit of statistical models was evaluated by *R*^2^ and RMSE. As a quantitative measure of the uncertainty, the observation-based 95% CI was determined [40].

(1) $$E=1-\Delta {\mathrm{OD}}_{t}/\Delta {\mathrm{OD}}_{c}$$

(2) $$\;E=1-\exp\left(-\exp\left(a+b\;\times {\;\log}_{10}\left(C\right)\right)\right)$$

where ΔOD_t_ is the absorbance change of treats from 0 to 15 min, ΔOD_c_ is the absorbance change of controls from 0 to 15 min, *E* is the effect, *C* is the concentration, *a* is location parameter, and *b* is slope parameter.

### 3.3. Experimental Design and Toxicity Evaluation of Mixtures

**Figure 5:**
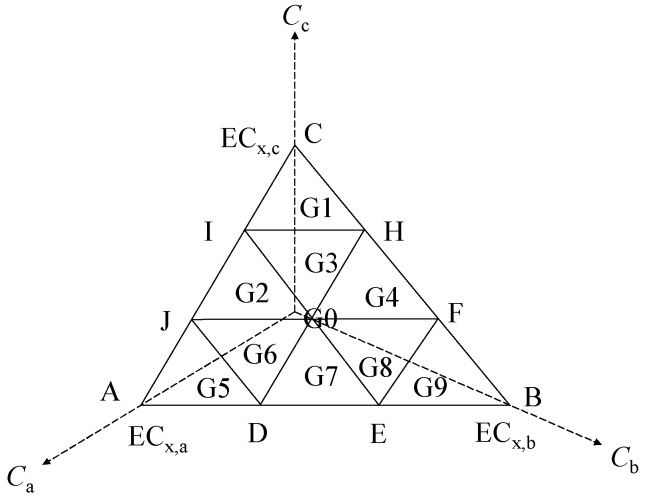
Equipartition equivalent-surface design (EESD) for ternary mixture.

Based on the EESD method shown in Figure 5 proposed by us recently [41], we constructed 10 ternary mixtures of G0–G9 with the toxic unit (EC_50_) ratios (1:1:1, 1:1:7, 4:1:4, 2:2:5, 1:4:4, 7:1:1, 5:2:2, 4:4:1, 2:5:2, 1:7:1). Each of the corresponding points was the center of gravity of the triangle. The 15 binary mixtures (B1–B15) were designed using the EquRay design [42], every two components were mixed according to toxicity unit (EC_50_) ratios of 1:5, 2:4, 3:3, 4:2, and 5:1. Single ILs assay suggested 3 EC*_x_* vertices of the ES on three concentration axis. These 28 equivalent-effect points were used to construct the mixture observed *x*%-effect ES by using the triangle-based cubic interpolation [41].

The models of CA (Equation (3)) and IA (Equation (4)) [3] were used to predict the CRC and ES of mixtures. For CRC, when the mixture predicted CRC was located within the CI of mixture observed CRC, the mixture presented additive action. When the mixture predicted CRC was located on the left or right of mixture observed CRC CI, the mixture presented antagonistic or synergistic action, respectively.

For ES, when the additive space composed of CA ES and IA ES was located within the CI of mixture observed ES, the mixture presented additive action. When the additive space was located below or above the CI of mixture observed ES, the mixture presented antagonistic or synergistic action, respectively.

The final expression form of CTC as shown in Equation (5) can be obtained in the present study by summarizing the derivation process of CTC in ref. [5]. It can be seen that CTC was independent of the selected standard reagent, and equivalent to the form of CA deformation expression. The judgment criteria in most of references were as follows: 80 ≤ CTC ≤ 120 indicated an additive action of the mixture, CTC > 120 indicated a synergistic action of the mixture, and CTC < 80 indicated an antagonistic action of the mixture [31]. 

In order to more accurately evaluate the toxic interactions of mixtures, we developed a CTC integrated with CI method (CTCICI) based on mixture observed EC*_x_* and its 95% CI in this study. In this situation, when 100 was included in the CI of mixture CTC, the mixture presented additive action. When the CI of mixture CTC was greater or smaller than 100, the mixture presented synergistic or antagonistic action, respectively.

(3) $$\sum_{i=1}^{n}\left(C_{i}/{\mathrm{EC}}_{x,i}\right)=1$$

(4) $$E_{\mathrm{mix}}=1-\overset{n}{\underset{i=1}{\prod }}\left(1-E_{i}\right)\;$$

(5) $$\mathrm{CTC}=100/\left({\mathrm{EC}}_{x,\mathrm{mix}}\;\times \;\sum_{i=1}^{n}\left(P_{i}/{\mathrm{EC}}_{x,i}\right)\right)$$

where *n* is the number of mixture components, *EC_x_*_,*i*_ is the concentration of *i*th component eliciting *x*% effect, *C_i_* is the concentration of the *i*th component in the mixture eliciting *x*% effect, *EC_x_*_,mix_ is the concentration of a mixture eliciting *x*% effect, *P_i_* is the concentration proportion of *i*th component in a mixture, *E*_mix_ is mixture effect, *E_i_* is the effect of the *i*th component in a mixture.

## 4. Conclusions

Prediction of the mixture toxicity of three ILs of [BMIM]BF_4_, [BMIM]PF_6_, and [HMIM]BF_4_ on acetylcholinesterase was accomplished by the concentration addition (CA), and independent action (IA) models. The equivalent-surface (ES) method was successfully used to evaluate the toxic interaction of mixtures. These mixtures were basically all additive action. It was of interest to observe the linear and biphasic relationship between the binary mixture toxicities and the concentration proportions of single ILs. This study demonstrated that the ES method can be used to evaluate the toxicity of ternary mixtures including ILs.

## Figures and Tables

**Table 1:** Concentration–response model of single ILs and their ternary mixtures (G0–G9) and binary mixtures (B1–B15) inhibiting AChE and related parameters

Toxicants	*P*([BMIM]BF_4_)	*P*([BMIM]PF_6_)	*P*([HMIM]BF_4_)	TU Ratio	C_0_	*a*	*b*	*R* ^2^	RMSE	EC_20_	EC_50_	EC_80_
[BMIM]BF_4_					9.36 × 10^−3^	5.612	1.685	0.994	0.030	6.02 × 10^−5^	2.83 × 10^−4^	8.95 × 10^−4^
[BMIM]PF_6_					7.92 × 10^−3^	5.383	1.638	0.993	0.031	6.28 × 10^−5^	3.09 × 10^−4^	1.01 × 10^−3^
[HMIM]BF_4_					8.70 × 10^−3^	5.678	1.688	0.999	0.012	5.59 × 10^−5^	2.62 × 10^−4^	8.28 × 10^−4^
G0	3.32 × 10^−1^	3.61 × 10^−1^	3.06 × 10^−1^	1:1:1	8.60 × 10^−3^	5.822	1.750	0.998	0.018	6.55 × 10^−5^	2.91 × 10^−4^	8.81 × 10^−4^
G1	1.17 × 10^−1^	1.27 × 10^−1^	7.56 × 10^−1^	1:1:7	8.66 × 10^−3^	5.639	1.657	0.999	0.012	4.92 × 10^−5^	2.38 × 10^−4^	7.66 × 10^−4^
G2	4.56 × 10^−1^	1.24 × 10^−1^	4.20 × 10^−1^	4:1:4	8.88 × 10^−3^	5.508	1.622	0.999	0.014	4.78 × 10^−5^	2.39 × 10^−4^	7.90 × 10^−4^
G3	2.28 × 10^−1^	2.47 × 10^−1^	5.25 × 10^−1^	2:2:5	8.63 × 10^−3^	6.003	1.791	0.999	0.015	6.47 × 10^−5^	2.78 × 10^−4^	8.20 × 10^−4^
G4	1.11 × 10^−1^	4.81 × 10^−1^	4.08 × 10^−1^	1:4:4	8.37 × 10^−3^	5.488	1.632	0.998	0.018	5.23 × 10^−5^	2.59 × 10^−4^	8.49 × 10^−4^
G5	7.77 × 10^−1^	1.21 × 10^−1^	1.02 × 10^−1^	7:1:1	9.09 × 10^−3^	5.446	1.607	0.997	0.019	4.76 × 10^−5^	2.42 × 10^−4^	8.08 × 10^−4^
G6	5.55 × 10^−1^	2.41 × 10^−1^	2.04 × 10^−1^	5:2:2	8.84 × 10^−3^	5.327	1.570	0.997	0.019	4.48 × 10^−5^	2.36 × 10^−4^	8.13 × 10^−4^
G7	4.32 × 10^−1^	4.69 × 10^−1^	9.94 × 10^−2^	4:4:1	8.56 × 10^−3^	5.259	1.560	0.998	0.017	4.65 × 10^−5^	2.48 × 10^−4^	8.59 × 10^−4^
G8	2.16 × 10^−1^	5.86 × 10^−1^	1.99 × 10^−1^	2:5:2	8.34 × 10^−3^	4.985	1.469	0.997	0.019	3.85 × 10^−5^	2.28 × 10^−4^	8.52 × 10^−4^
G9	1.05 × 10^−1^	7.98 × 10^−1^	9.67 × 10^−2^	1:7:1	8.12 × 10^−3^	5.212	1.559	0.996	0.022	4.95 × 10^−5^	2.64 × 10^−4^	9.16 × 10^−4^
B1	1.55 × 10^−1^	8.45 × 10^−1^	0	1:5:0	8.11 × 10^−3^	5.185	1.554	0.996	0.023	4.99 × 10^−5^	2.68 × 10^−4^	9.33 × 10^−4^
B2	3.15 × 10^−1^	6.85 × 10^−1^	0	2:4:0	8.32 × 10^−3^	5.170	1.547	0.997	0.022	4.88 × 10^−5^	2.64 × 10^−4^	9.24 × 10^−4^
B3	4.79 × 10^−1^	5.21 × 10^−1^	0	3:3:0	8.55 × 10^−3^	5.041	1.493	0.998	0.018	4.16 × 10^−5^	2.39 × 10^−4^	8.76 × 10^−4^
B4	6.48 × 10^−1^	3.52 × 10^−1^	0	4:2:0	8.80 × 10^−3^	5.106	1.510	0.996	0.022	4.22 × 10^−5^	2.38 × 10^−4^	8.58 × 10^−4^
B5	8.22 × 10^−1^	1.78 × 10^−1^	0	5:1:0	9.07 × 10^−3^	5.774	1.729	0.996	0.026	6.21 × 10^−5^	2.81 × 10^−4^	8.62 × 10^−4^
B6	1.78 × 10^−1^	0	8.22 × 10^−1^	1:0:5	8.81 × 10^−3^	5.862	1.701	0.999	0.012	4.70 × 10^−5^	2.18 × 10^−4^	6.82 × 10^−4^
B7	3.52 × 10^−1^	0	6.48 × 10^−1^	2:0:4	8.92 × 10^−3^	5.894	1.719	0.999	0.012	5.00 × 10^−5^	2.28 × 10^−4^	7.05 × 10^−4^
B8	5.20 × 10^−1^	0	4.80 × 10^−1^	3:0:3	9.03 × 10^−3^	5.600	1.632	0.999	0.014	4.46 × 10^−5^	2.21 × 10^−4^	7.25 × 10^−4^
B9	6.85 × 10^−1^	0	3.15 × 10^−1^	4:0:2	9.14 × 10^−3^	6.067	1.803	0.998	0.019	6.36 × 10^−5^	2.70 × 10^−4^	7.92 × 10^−4^
B10	8.44 × 10^−1^	0	1.56 × 10^−1^	5:0:1	9.25 × 10^−3^	5.355	1.577	0.998	0.018	4.50 × 10^−5^	2.35 × 10^−4^	8.06 × 10^−4^
B11	0	1.91 × 10^−1^	8.09 × 10^−1^	0:1:5	8.54 × 10^−3^	5.545	1.618	0.998	0.016	4.42 × 10^−5^	2.22 × 10^−4^	7.36 × 10^−4^
B12	0	3.71 × 10^−1^	6.29 × 10^−1^	0:2:4	8.39 × 10^−3^	5.491	1.616	0.999	0.010	4.72 × 10^−5^	2.37 × 10^−4^	7.88 × 10^−4^
B13	0	5.41 × 10^−1^	4.59 × 10^−1^	0:3:3	8.26 × 10^−3^	5.617	1.672	0.998	0.018	5.54 × 10^−5^	2.64 × 10^−4^	8.42 × 10^−4^
B14	0	7.02 × 10^−1^	2.98 × 10^−1^	0:4:2	8.14 × 10^−3^	5.323	1.589	0.997	0.019	5.08 × 10^−5^	2.63 × 10^−4^	8.90 × 10^−4^
B15	0	8.55 × 10^−1^	1.45 × 10^−1^	0:5:1	8.02 × 10^−3^	4.981	1.487	0.998	0.018	4.38 × 10^−5^	2.53 × 10^−4^	9.34 × 10^−4^

Note: *P_i_* is concentration proportion of components in mixtures; TU is the toxic unit by EC_50_; C_0_ is stock concentration; *a* is location parameter; *b* is slope parameter; *R*^2^ is coefficient of determination; RMSE is root-mean-square error; EC_80_, EC_50_, and EC_20_ are the 80%, 50%, 20%-effect concentration, respectively; all the units of C_0_, EC_80_, EC_50_, and EC_20_ are mol/L; Integer 0 in *P_i_* and TU ratio means that the corresponding component does not exist.

**Table 2:** Joint toxicity effect of [BMIM]BF_4_, [BMIM]PF_6_, and [HMIM]BF_4_ on AChE.

	E = 20%				E = 50%				E = 80%			
Mixtures	CTC	CTC_UL_	CTC_LL_	Interaction	CTC	CTC_UL_	CTC_LL_	Interaction	CTC	CTC_UL_	CTC_LL_	Interaction
G0	91	131	68	additive	98	120	88	additive	103	113	97	additive
G1	116	149	93	additive	114	127	107	synergistic	112	121	94	additive
G2	123	165	95	additive	116	132	106	synergistic	111	114	98	additive
G3	90	121	71	additive	100	120	95	additive	108	119	85	additive
G4	114	167	83	additive	110	133	97	additive	108	121	97	additive
G5	126	193	89	additive	117	140	102	synergistic	111	121	96	additive
G6	134	204	94	additive	120	144	104	synergistic	111	123	96	additive
G7	131	192	95	additive	118	141	103	synergistic	109	123	97	additive
G8	158	248	109	synergistic	129	158	108	synergistic	111	131	93	additive
G9	125	209	83	additive	114	146	93	additive	107	131	86	additive
B1	125	213	82	additive	114	147	92	additive	106	133	85	additive
B2	127	211	85	additive	114	145	93	additive	105	129	86	additive
B3	148	226	104	synergistic	124	150	105	synergistic	109	127	93	additive
B4	145	246	95	additive	123	154	100	additive	109	129	89	additive
B5	98	170	64	additive	102	131	85	additive	106	118	92	additive
B6	121	152	98	additive	122	130	115	synergistic	123	130	104	synergistic
B7	115	145	93	additive	118	127	113	synergistic	121	127	102	synergistic
B8	130	175	100	additive	124	137	112	synergistic	119	130	99	additive
B9	93	134	69	additive	102	121	98	additive	110	124	85	additive
B10	132	201	94	additive	119	141	103	synergistic	110	119	93	additive
B11	129	182	97	additive	122	140	110	synergistic	117	120	100	additive
B12	124	152	102	synergistic	117	130	110	synergistic	113	115	104	synergistic
B13	107	156	78	additive	108	131	95	additive	109	121	99	additive
B14	119	183	84	additive	112	138	95	additive	107	126	92	additive
B15	141	213	99	additive	119	146	100	additive	105	127	88	additive

Note: CTC: co-toxicity coefficient; CTC_LL_: the lower limit of mixture CTC CI; CTC_UL_: the upper limit of mixture CTC CI.

**Table 3:** Information about chemicals used in the experiment.

Chemicals	Molecular Structure	Source	Purity	CAS No.	Molecular Weight
AChE	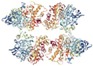	Sigma	217 units/mg protein	9000-81-1	−320 kDa (tetramer)
ATCI	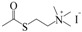	Aladdin	98%	1866-15-5	289.18
DTNB	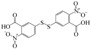	Vetec	98%	69-78-3	396.35
[BMIM]BF_4_	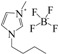	CGCC	>99%	174501-65-6	225.89
[BMIM]PF_6_	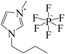	CGCC	>99%	174501-64-5	284.18
[HMIM]BF_4_	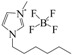	CGCC	>99%	244193-50-8	254.08

Note: The molecular structure of acetylcholinesterase (*Electrophorus electricus*) was from the website www.rcsb.org/3d-view/1C2O/1.
